# Supplementary material for: A CRISPR-Cas9 screen identifies EXO1 as a formaldehyde resistance gene
Source: Nat Commun. 2023 Jan 24;14:381. doi: 10.1038/s41467-023-35802-y (PMC9873647; doi:10.1038/s41467-023-35802-y)
Supplement: Supplementary file 1 — Supplementary Information [file 41467_2023_35802_MOESM1_ESM.pdf]

SUPPLEMENTARY INFORMATION

A CRISPR-Cas9 screen identifies EXO1 as a formaldehyde resistance gene

Yuandi Gao, Laure Guitton-Sert, Julien Dessapt, Yan Coulombe, Amélie Rodrigue, Larissa Milano, Andréanne Blondeau, Nicolai Balle Larsen, Julien P. Duxin, Samer Hussein, Amélie Fradet-Turcotte, and Jean-Yves Masson

Supplementary Table 1. List of antibodies used in this study

Antibodies	Source	Dilution	Identifier
Anti-Exonuclease 1	Abcam	1:1000 (WB)	ab95068
Phospho-Chk1 (Ser345) (133D3) Rabbit mAb	Cell Signaling Technology	1:1000 (WB)	2348T
Chk1 antibody [DCS-310]	GeneTex	1:1000 (WB)	GTX42610
RPA2 (p Ser4, p Ser8)	Novus	1:1000 (WB)	NBP1-23017
RPA70	Calbiochem	1:1000 (WB)	NA-13
ADH5	Proteintech	1:1000 (WB)	11051-1-AP
ALDH2	Proteintech	1:2000 (WB)	15310-1-AP
anti-α tubulin	Abcam	1:100000 (WB)	ab7291
Monoclonal Anti-β-Actin	Sigma	1:10000 (WB)	A5316
Vinculin monoclonal antibody	Sigma	1:100000 (WB)	V9131
Peroxidase-AffiniPure Goat Anti-Rabbit IgG (H+L)	Cedarlane Labs	1:10000 (WB)	111-035-144
Peroxidase-AffiniPure Sheep Anti-Mouse IgG (H+L)	Cedarlane Labs	1:10000 (WB)	515-035-062
γH2AX	EMD Millipore	1:5000 (IF)	05636
pS1778-p53BP1	Cell signaling technology	1:400 (IF)	26755
Alexa Fluor 568 goat anti-rabbit	Thermo Fisher Scientific	1:1000 (IF)	A-11011
Alexa Fluor 488 goat anti-mouse	Thermo Fisher Scientific	1:1000 (IF)	A-11001
anti-RAD51	B-bridge International	1:5000 (IF)	70–001
anti-cyclin A	BD Biosciences	1:400 (IF)	611268
53BP1	Novus	1:1000 (IF)	100304
RPA34-19	Calbiochem	1:400 (IF)	NA18
pS714-pEXO1	from Dr. Kum Kum Khanna	1:200 (IF)	
anti-Topoisomerase I-DNA Covalent Complexes Antibody, clone 1.1A	Millipore-Sigma	1:250 (IF)	MABE1084
anti-Geminin	Proteintech	1:500 (IF)	10802-1-AP
FANCD2	Novus	1 :1000(IF)	NB100-182
anti-Geminin	Abcam	1 :2000(IF)	ab104306
anti-pS2056-pDNAPKcs	Abcam	1:1000 (IF)	ab18192
Anti-BrdU antibody [BU1/75 (ICR1)]	Abcam	1:20 (combing)	ab6326
Anti BrdU	Beckton Dickinson	1:20 (combing)	347580
anti-Rat Alexa 568	Thermo Fisher Scientific	1:50 (combing)	A11077

anti-Mouse IgG1 Alexa 488	Thermo Fisher Scientific	1:50 (combing)	A21121
anti-ssDNA	Millipore	1:50 (combing)	MAB3868
Goat anti Mouse IgG2a Secondary Antibody, Alexa Fluor 647	Thermo Fisher Scientific	1:50 (combing)	A21241

Supplementary Table 2. Reagents or resources used in this study

Reagents or resources	Source	Identifier
DMEM high glucose	Thermo Fisher Scientific	10063542
DMEM/F-12	Thermo Fisher Scientific	11320033
Fetal Bovine serum	Gibco	12483-020
Trypsin	Sigma	T4049-500ML
PBS 1X, PH 7.4	Wisent Bio Products	311-010-CL
HEPES (1M)	Life Technologies	15630080
Polybrene	Millipore-Sigma	H9268
formaldehyde	J.T.Baker	CAJT2106-1
Mitomycin C	Sigma	M0440-5MG
Alt-R CRISPR-Cas9 EXO1 crRNA	IDT	Hs.Cas9.EXO1.1.AC
Alt-R CRISPR-Cas9 tracrRNA	IDT	1073189
Alt-R S.p. HIFI Cas9 nuclease V3	IDT	1081060
Gene Knockout kit v2	Synthego	human EXO1
nocodazole	Sigma	M1404-2MG
Hoechst 33342	Invitrogen life technology	H3570
Propidium iodide	Sigma-Aldrich	P4170-10MG
4,5′,8-Trimethylpsoralen	Sigma	T6137
Angelicin	Sigma	A0956
G418 sulfate	Wisent INC/Multicell	400-130-QL
Hygromycin B (50 mg/mL)	Thermo Fisher Scientific	10687010
beta-Agarase I	New England Biology	M0392L
Proteinase K	Thermo Fisher Scientific	BP1700500
Monarch® RNaseA	New England Biology	T3018L
BSA	Sigma	A7906
Bio-Rad Protein Assay Dye Reagent	BIO-RAD	5000006
ProLong® Gold Antifade Mountant	Invitrogen life technology	P-36930
ProLong® Gold Antifade Mountant with DAPI	Invitrogen life technology	P-36931
Anti-FLAG M2 Affinity Gel	Sigma-Aldrich	A2220
TALON® Metal Affinity Resin	Clontech	635504
Benzonase nuclease	Thermo Fisher Scientific	707463
4-12% Bis-Tris gel	Life Technologies	NW04122BOX
NuPAGE™ 3 to 8%, Tris-Acetate, 1.0–1.5 mm, Mini Protein Gels	Thermo Fisher Scientific	EA0375PK2
**Critical Commercial Assays**		
KAPA HiFi HS RM	Roche	07958935001
QiAamp Blood Maxi Kit	Qiagen	51194
QIAquick PCR purification kit	Qiagen	28104
QIAquick gel extraction kit	Qiagen	28706
Lipofectamine RNAiMAX transfection reagent	Invitrogen life technology	13778150
Lipofectamine 2000 transfection reagent	Invitrogen life technology	11668019

Click-iT™ EdU Cell Proliferation Kit for Imaging, Alexa Fluor™ 647 dye	Thermo Fisher Scientific	C10340
Q5 Site-Directed Mutagenesis Kit	New England Biolabs	E0554S
Bac to Bac Expression System	Invitrogen	10359016
Quant-iT™ PicoGreen™ dsDNA Assay Kit	Thermo Fisher Scientific	P7589
SilverQuest™ Silver Staining Kit	Invitrogen	LC6070
**Software and Algorithms**		
DrugZ	DrugZ algorithm	V1.1.0.2
Prism	GraphPad	Version 7
CellProfiler	CellProfiler Software	V3.1.8
Image Reader FLA-5000	FLA-5100 phosphorimager Software	V1.0
Gen5 Data Analysis Software V3.03	Gen5 Data Analysis Software	V3.03
iBright FL1500 Imaging System	iBright Analysis Software	V5.0.1

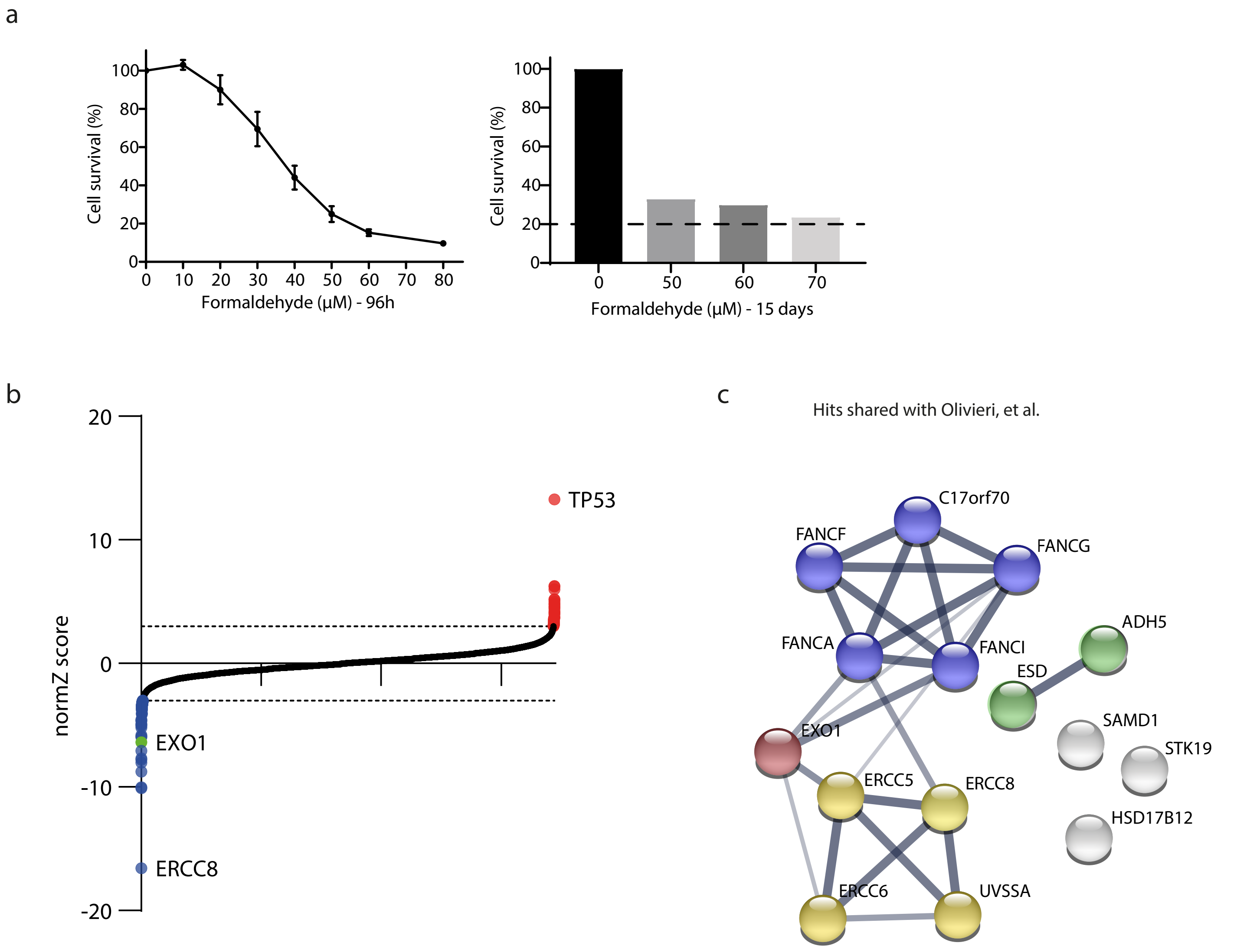

Supplementary Figure 1
(a) Left. Survival curve of RPE-1 WT cells under the indicated concentration of formaldehyde treatment for 96 h. Right. Cell survival percentage of RPE-1 WT cells with 0, 50, 60, 70 μM formaldehyde treatment for 15 days. Cells treated with 70 μM had approximately 20% survival at the end of the 15-day treatment.
(b) NormZ score for all the genes in the CRISPR-Cas9 screen. Dashed lines represent significance thresholds. Red and blue dots correspond to genes which corresponding sgRNAs are significantly increased and decreased respectively. (c) Shared hits from our CRISPR-Cas9 screen and A Genetic Map of the Response to DNA Damage in Human Cells (Cell 182, 481-496 e421), presented by STRING (green: formaldehyde catabolism genes, blue: Fanconi Anemia pathway, Yellow: Transcription –coupled Nuclear Excision Repair, red: EXO1).

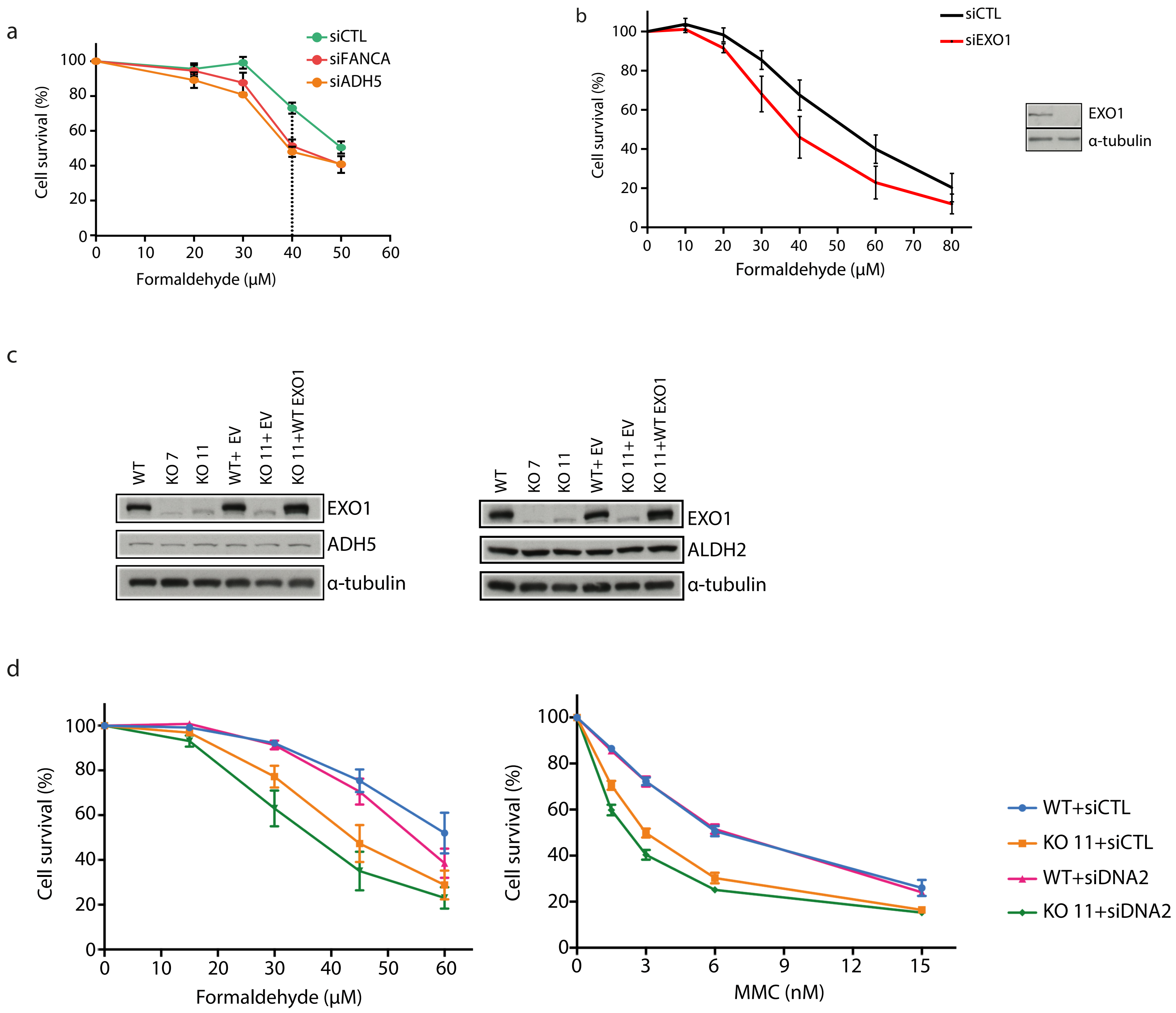

Supplementary Figure 2

(a) Survival curve of RPE-1 WT cells transfected with siCTL, siFANCA or siADH5 under different concentrations of formaldehyde treatment for 96 h. From this curve, 40 μM of formaldehyde was chosen for the siRNA screen. Data is presented with ± SEM from 3 independent experiments.

(b) Survival curve of RPE-1 WT cells transfected with siCTL or siEXO1 and treated with different formaldehyde concentrations for 96 h. EXO1 protein levels in siCTL and siEXO1 transfected cells, with α-tubulin as the loading control, were monitored by western blotting. Data is presented with ± SEM from 3 independent experiments.

(c) Protein levels of EXO1, ADH5 and ALDH2 in RPE-1 WT, EXO1 KO7, EXO1 KO11 and AAVS1 complemented cells. Western blotting against α-tubulin was used as a loading control.

(d) Survival curve of RPE-1 WT or EXO1 KO11 cells transfected with siCTL or siDNA2 under different concentrations of formaldehyde or MMC treatment for 96 h. Data is presented with ± SEM from 3 independent experiments.

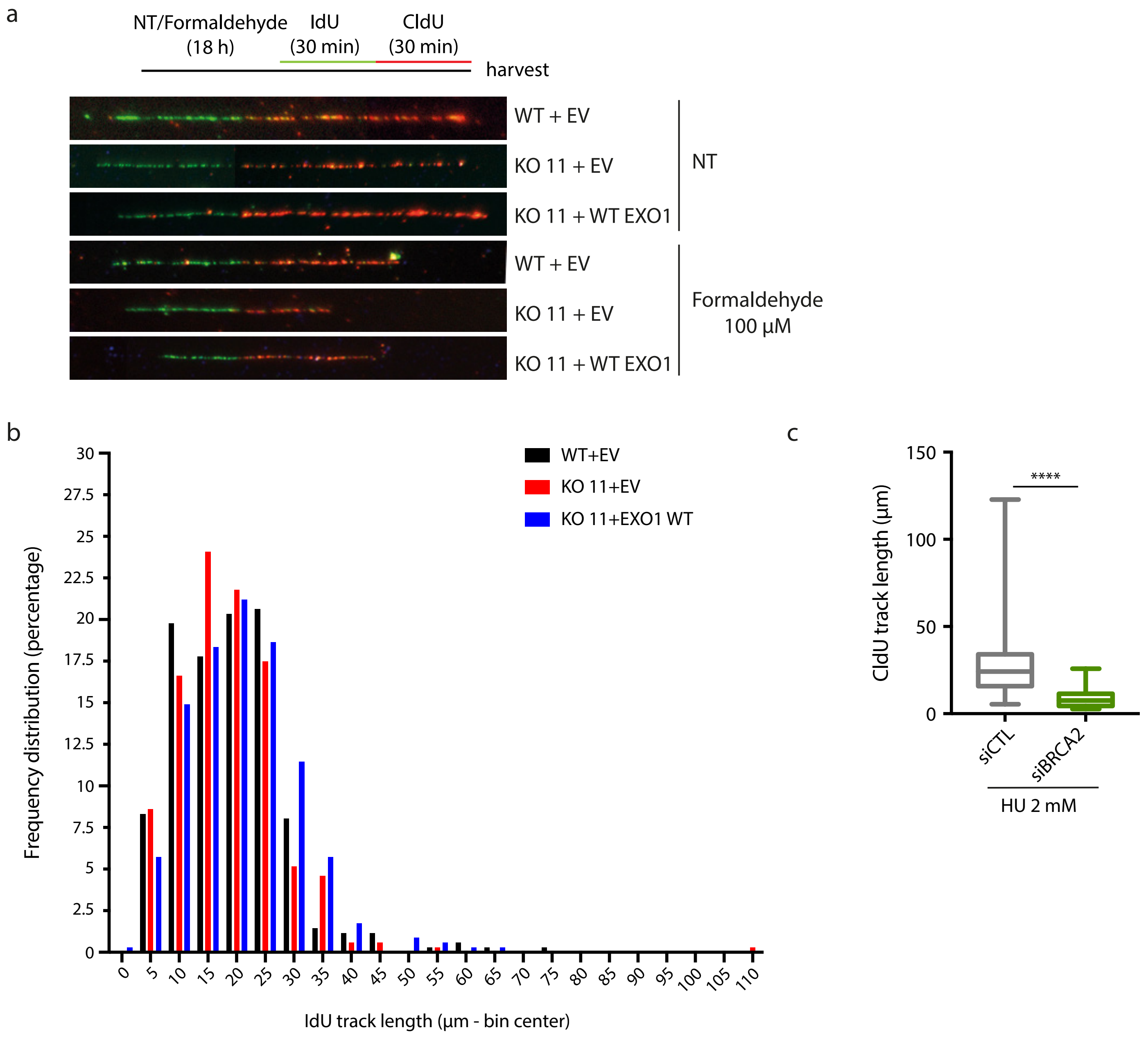

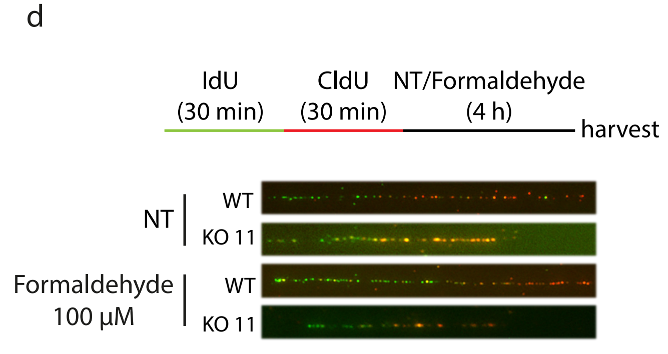

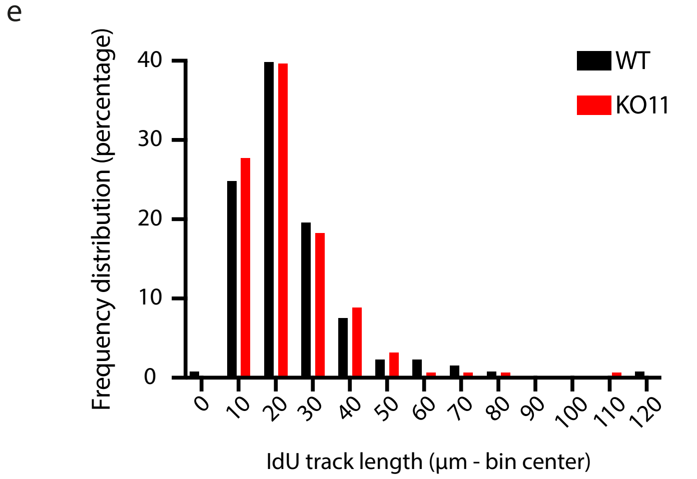

Supplementary Figure 3

(a) Schematics and representative pictures for Figure 3b.

(b) IdU track length distribution in RPE-1 EXO1 WT with AAVS1 empty vector (WT + EV) cells, KO 11 with AAVS1 empty vector (KO 11+EV) cells and KO 11 complemented with WT EXO1 (KO 11+WT EXO1) cells without treatment, corresponding to NT in Figure 3b. (c) CldU track length of DNA fibers from RPE-1 WT cells transfected with siCTL or siBRCA2, and treated with 2 mM hydroxyurea (HU) for 4 h. Data is presented with ± SEM from 3 independent experiments. ****$p < 0.0001$ (Mann-Whitney test). (d) Schematics and representative pictures for Figure 3c.

(e) IdU track length distribution in RPE-1 WT and EXO1 KO 11 cells related to Figure 3c.

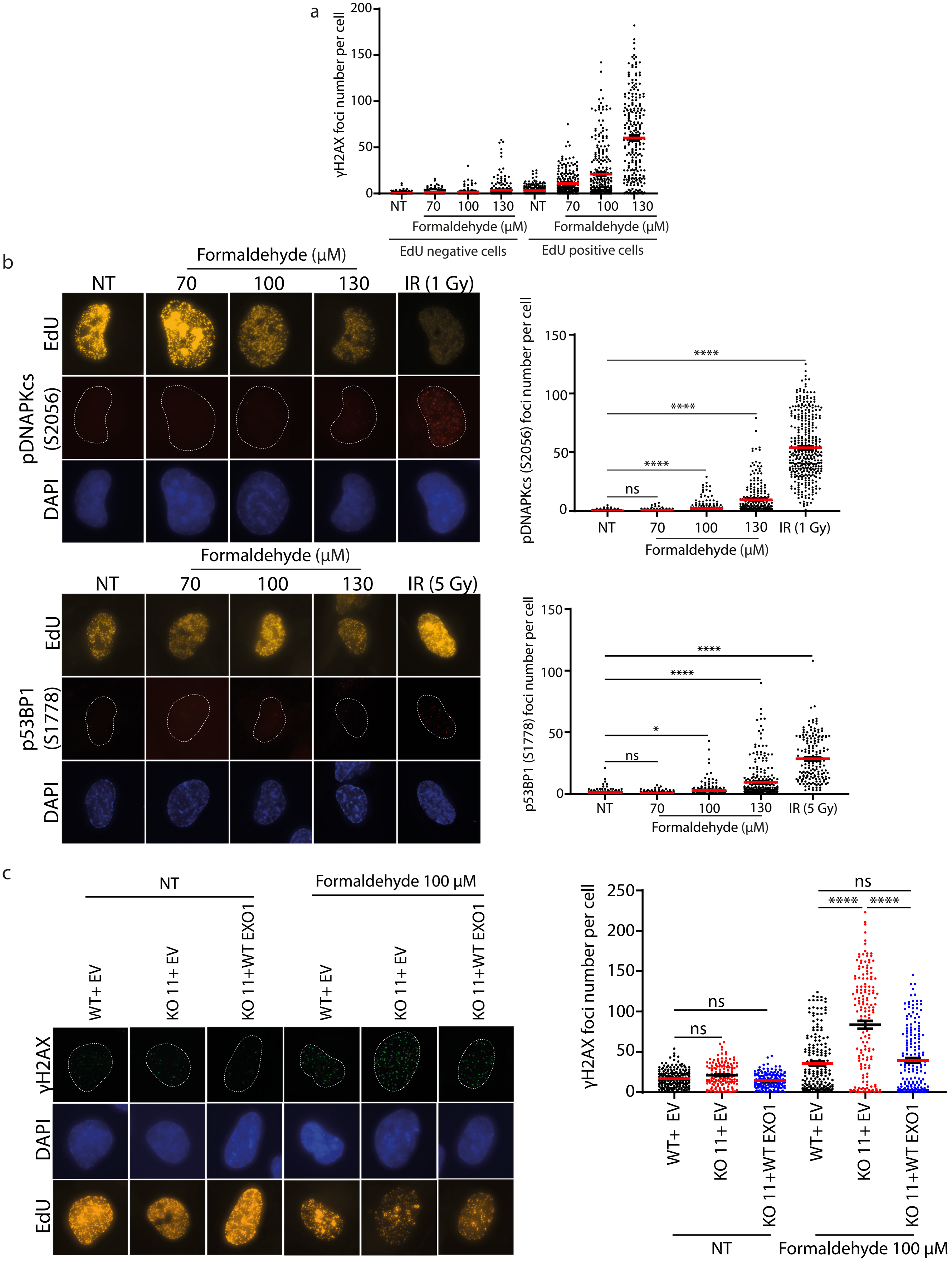

Supplementary Figure 4

(a) Quantification of the number of γH2AX (pSer 139) foci in EdU negative or positive RPE-1 WT cells treated with 0, 70, 100, and 130 μM formaldehyde for 18 h. (b) Immunofluorescence staining against pDNAPKcs (pSer 2056) and p53BP1 (pSer 1778) in S-phase RPE-1 WT cells determined by EdU staining under 0, 70, 100, and 130 μM formaldehyde for 18 h. Data is shown with mean ± SEM. ns: non-significant, *p<0.1 and ****p < 0.0001 (One-way ANOVA, followed by Kruskal-Wallis test). (c) Immunofluorescence staining of γH2AX (pSer 139) in AAVS1 complemented cells with or without 100 μM formaldehyde treatment for 18 h. Data is shown with mean ± SEM. ns: non-significant, ****p < 0.0001 (One-way ANOVA, followed by Kruskal-Wallis test). EV: Empty Vector

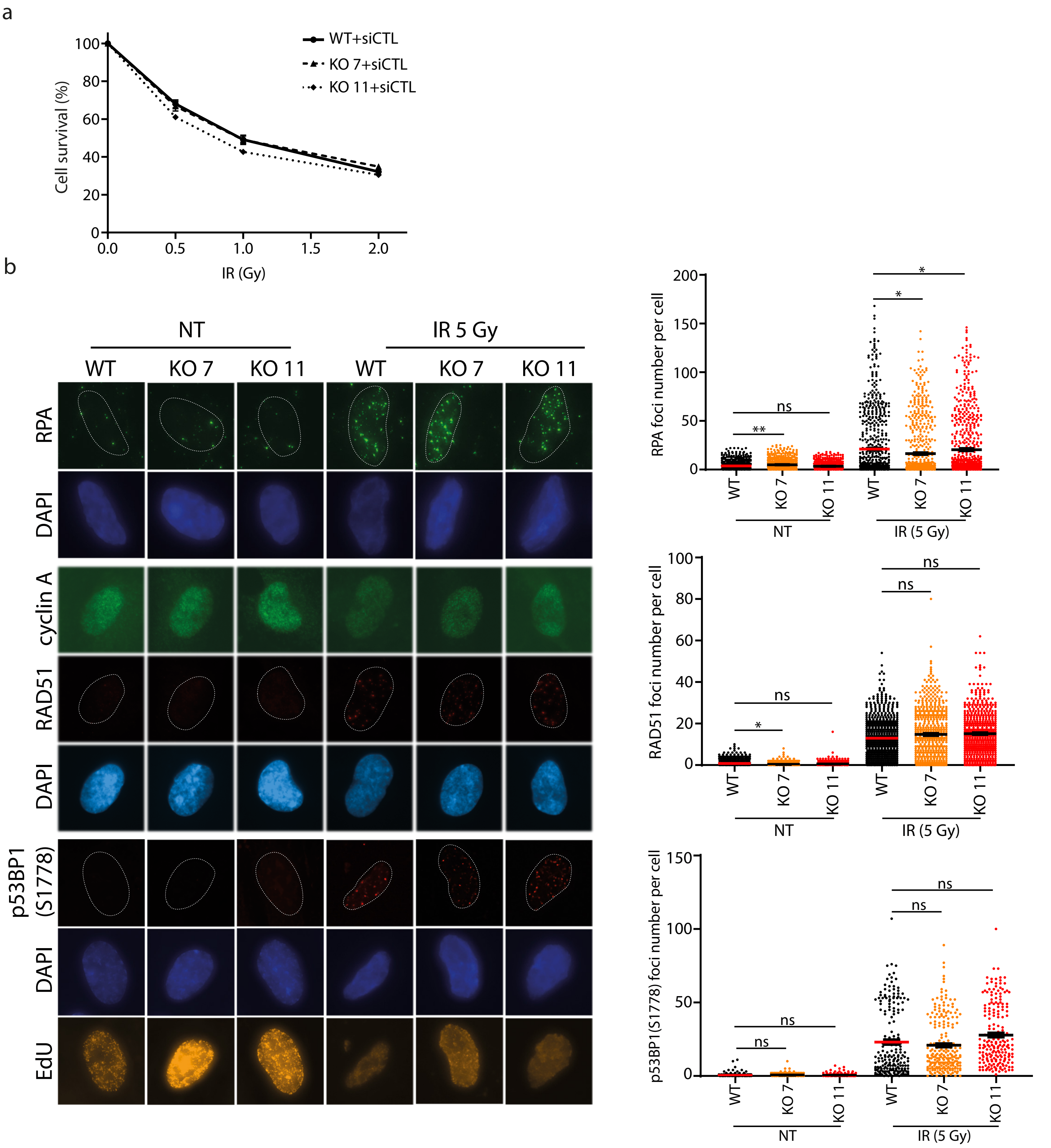

Supplementary Figure 5

(a) Survival curve of RPE-1 WT, EXO1 KO 7, or EXO1 KO11 transfected with siCTL and after irradiation (IR) (0.5-2 Gy). Cell survival was monitored 96 h after irradiation. Data is shown with mean ± SEM. (b) Immunofluorescence staining of RPA2, Cyclin A, RAD51, p53BP1 (pSer 1778) in RPE-1 WT, EXO1 KO 7, and EXO1 KO 11 cells without treatment or irradiated at 5 Gy (IR). Quantification was performed in cells in S-G2, or S phase, identified respectively by Cyclin A and EdU staining. Data is shown with mean ± SEM. ns: non-significant, *p<0.1, **p<0.01 (One-way ANOVA, followed by Kruskal-Wallis test).

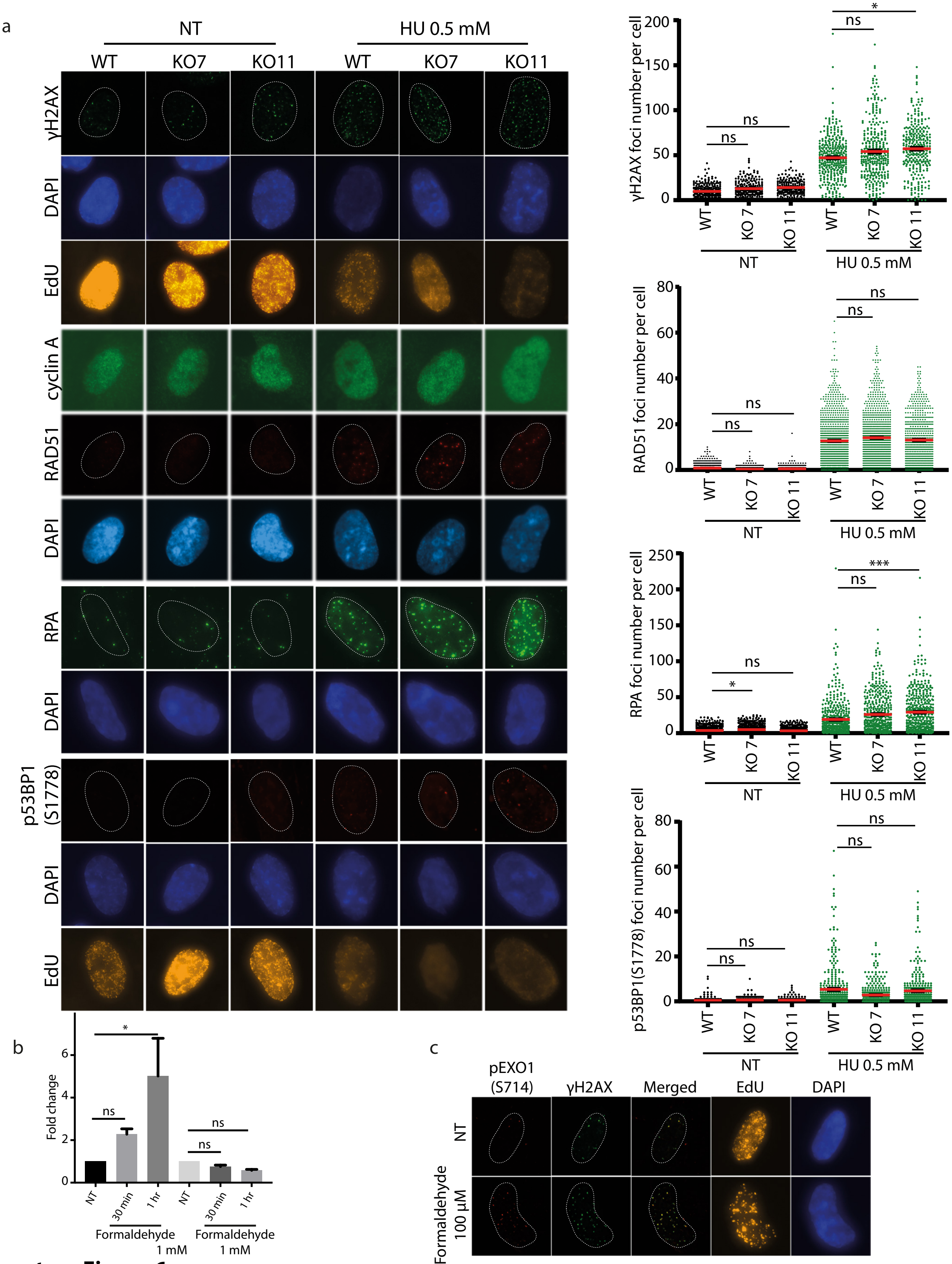

Supplementary Figure 6

(a) Immunofluorescence staining against γH2AX (pSer 139), Cyclin A, RAD51, RPA2, and p53BP1 (pSer 1778) in RPE-1 WT, EXO1 KO 7, and EXO1 KO 11 cells either mock-treated or treated with 0.5 mM HU for 18 h. Quantification was performed in cells in S-G2, or S phase, identified respectively by Cyclin A and EdU staining. Data is shown with mean ± SEM. ns: non-significant, *p<0.1 and ***p < 0.001 (One-way ANOVA, followed by Kruskal-Wallis test).

(b) Quantification of protein levels of EXO1 in RPE-1 WT cells with or without 1 mM formaldehyde treatment for 30 min or 1 h corresponding to the blots from 3 independent experiments with mean ± SEM. P-values are labeled accordingly to each condition, *p<0.1 (Ordinary One-way ANOVA). (c) Immunofluorescence staining against pEXO1 (pSer 714) and γH2AX (pSer 139) in S-phase (EdU positive cells) RPE-1 WT cells under 0 or 100 μM formaldehyde treatment for 18 h.

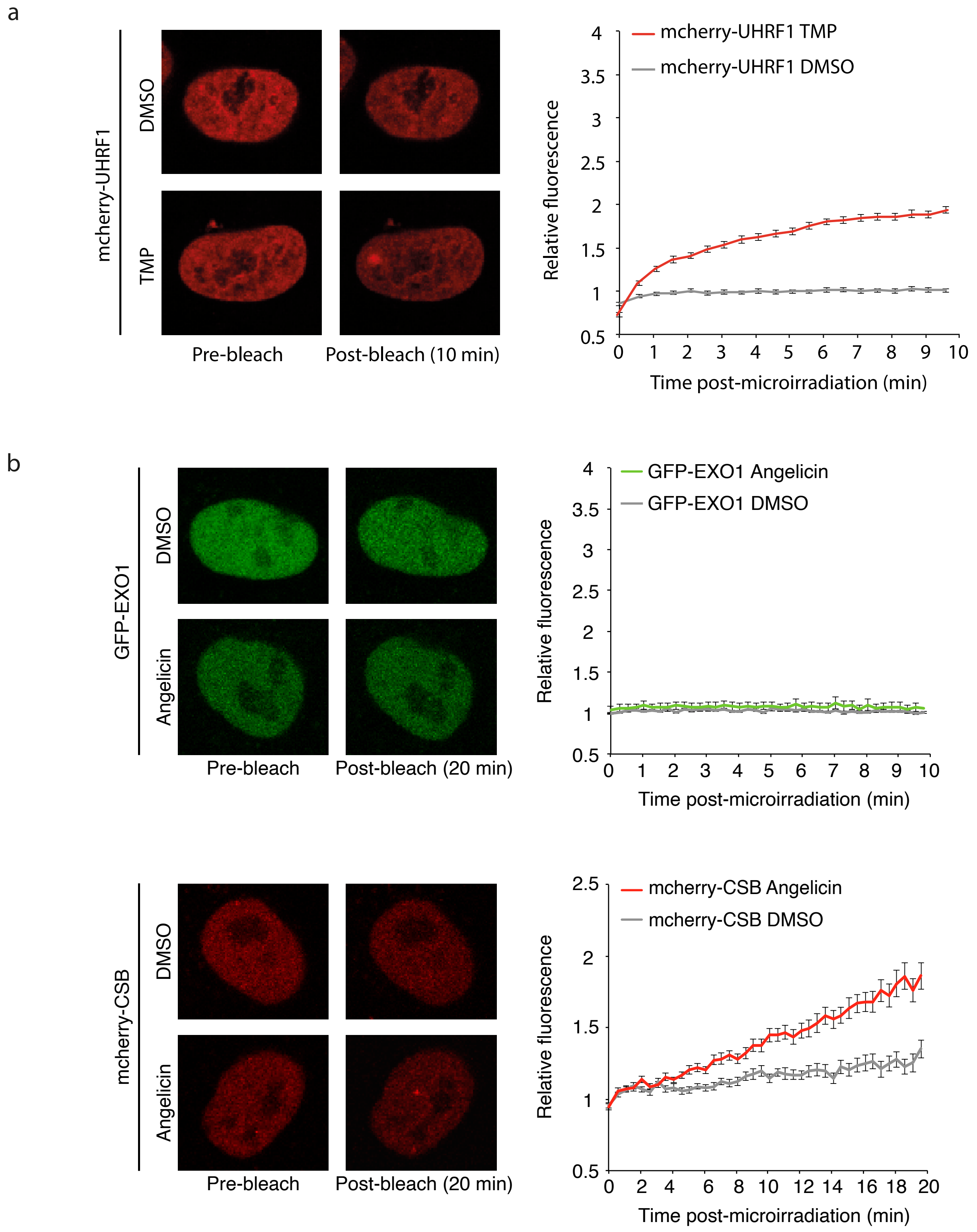

Supplementary Figure 7

(a) Quantitative evaluation of recruitment kinetics for mCherry-UHRF1 to ICL sites induced by TMP incubation followed by UV micro-irradiation in HeLa cells. Mean curves ± SEM are shown (n>100 cells per condition). (b) Quantitative evaluation of recruitment kinetics for GFP-EXO1 and mCherry-CSB to monoadducts induced by angelicin incubation followed by UV micro-irradiation in HeLa cells. Mean curves ± SEM are shown (n>100 cells per condition).

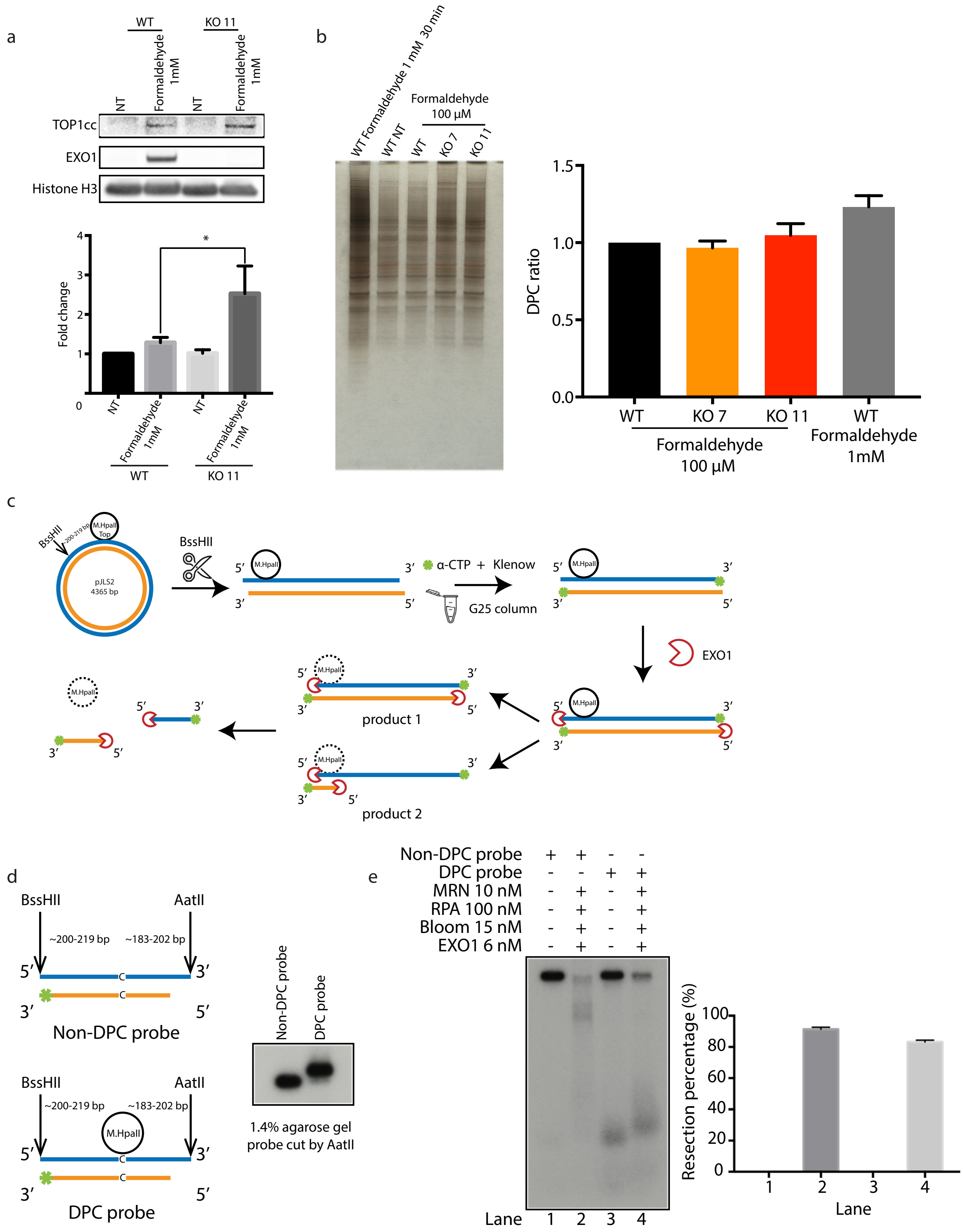

Supplementary Figure 8

a) Top. Protein levels of topoisomerase I-DNA covalent complexes (TOP1cc) and EXO1 in RPE-1 WT or EXO1 KO 11 cells with or without 1 mM formaldehyde treatment for 1 h in chromatin fraction (anti-Histone H3). Bottom. The quantification corresponds to the blots from 5 independent experiments with mean ± SEM. P-value is labelled *p<0.1 (Mann-Whitney test). (b) Left. RADAR assay of RPE-1 WT or EXO1 knockout cells challenged with formaldehyde. Right. Quantification of the smear intensity in 5 individual experiments. (c) Schematics of the M.HpaII DPC substrate, labelling strategy, and EXO1-mediated degradation products. The green asterisk denotes the position of the radiolabel. The “eppendorf” icon by Anthony Ledoux from thenounproject.com and the “scissors” icon by Kenny Sing from iconscout.com were used. (d) The integrity of the DPC probe was confirmed by cutting with AatII restriction enzyme. A shift of the DPC probe compared to non-DPC probe was detected via 1.4% agarose gel electrophoresis due to the crosslink of M.HpaII. (e) In vitro resection products of incubating puri¬fied MRN-RPA-BLM-EXO1 with non-DPC or DPC probes after agarose gel electrophoresis and detected by autoradiography. The percentage of resection was quantified.

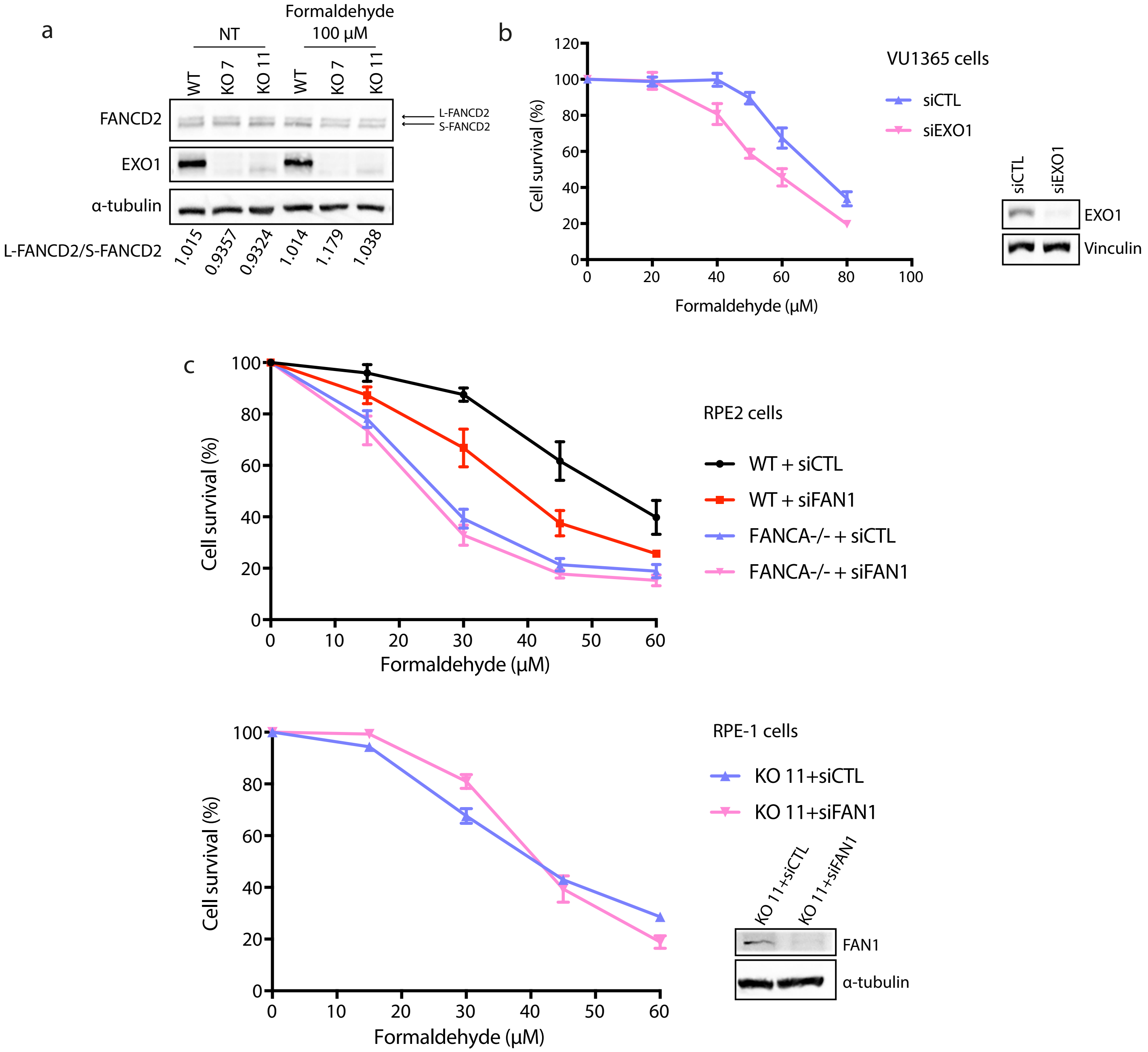

Supplementary Figure 9

(a) FANCD2 and EXO1 protein levels in RPE-1 WT, EXO1 KO 7 and EXO1 KO 11 cells treated with or without 100 μM formaldehyde for 18 h were monitored by western blotting. The ubiquitination level of FANCD2 is quantified using L-FANCD2/S-FANCD2 which is normalized by α-tubulin. Data presented is the mean from 4 independent experiments. (b) Left. Survival curve of VU1365 FANCA-/- cells transfected with siCTL or siEXO1 under different concentrations of formaldehyde treatment for 96 h. Data is presented with ± SEM from 3 independent experiments. Right. EXO1 protein levels with Vinculin as a loading control. (c) Top. Survival curve of RPE2 WT or RPE2 FANCA KO cells transfected with siCTL or siFAN1 and treated with different formaldehyde concentrations for 96 h. Bottom. Survival curve of RPE-1 WT or EXO1 KO 11 cells transfected with siCTL or siFAN1 and treated with different formaldehyde concentrations for 96 h. Data is presented with ± SEM from 3 independent experiments. Knockdown efficiency as measured by FAN1 protein levels is also shown.
