# Supplementary material for: A CRISPR-Cas9 screen identifies EXO1 as a formaldehyde resistance gene
Source: Nat Commun. 2023 Jan 24;14:381. doi: 10.1038/s41467-023-35802-y (PMC9873647; doi:10.1038/s41467-023-35802-y)
Supplement: Supplementary file 3 — Description of Additional Supplementary Files [file 41467_2023_35802_MOESM3_ESM.pdf]

Description of Additional Supplementary Files

File Name: Supplementary Data 1
Description: DrugZ analysis of the CRISPR-Cas screen

File Name: Supplementary Data 2
Description: Survival curves statistical analyses
