# Supplementary material for: A CRISPR-Cas9 screen identifies EXO1 as a formaldehyde resistance gene
Source: Nat Commun. 2023 Jan 24;14:381. doi: 10.1038/s41467-023-35802-y (PMC9873647; doi:10.1038/s41467-023-35802-y)
Supplement: Supplementary file 6 — Reporting Summary [file 41467_2023_35802_MOESM6_ESM.pdf]

nature portfolio

Corresponding author(s): Masson/NCOMMS-22-03197A

Last updated by author(s): Oct 12, 2022

Reporting Summary

Nature Portfolio wishes to improve the reproducibility of the work that we publish. This form provides structure for consistency and transparency in reporting. For further information on Nature Portfolio policies, see our Editorial Policies and the Editorial Policy Checklist.

Statistics

For all statistical analyses, confirm that the following items are present in the figure legend, table legend, main text, or Methods section.

n/a	Confirmed	
☐	☒	The exact sample size (*n*) for each experimental group/condition, given as a discrete number and unit of measurement
☐	☒	A statement on whether measurements were taken from distinct samples or whether the same sample was measured repeatedly
☐	☒	The statistical test(s) used AND whether they are one- or two-sided<br>*Only common tests should be described solely by name; describe more complex techniques in the Methods section.*
☒	☐	A description of all covariates tested
☒	☐	A description of any assumptions or corrections, such as tests of normality and adjustment for multiple comparisons
☐	☒	A full description of the statistical parameters including central tendency (e.g. means) or other basic estimates (e.g. regression coefficient) AND variation (e.g. standard deviation) or associated estimates of uncertainty (e.g. confidence intervals)
☒	☐	For null hypothesis testing, the test statistic (e.g. *F*, *t*, *r*) with confidence intervals, effect sizes, degrees of freedom and *P* value noted<br>*Give P values as exact values whenever suitable.*
☒	☐	For Bayesian analysis, information on the choice of priors and Markov chain Monte Carlo settings
☒	☐	For hierarchical and complex designs, identification of the appropriate level for tests and full reporting of outcomes
☒	☐	Estimates of effect sizes (e.g. Cohen's *d*, Pearson's *r*), indicating how they were calculated

Our web collection on statistics for biologists contains articles on many of the points above.

Software and code

Policy information about availability of computer code

Data collection	Raw sequencing result data files (i.e. FASTQ files) quality was assessed by FastQC analysis. MAGeCK count command 4 was used to identify library adapter length from FASTQ files, to perform primary alignment against the TKOv1 library, and to provide sgRNA read counts. Once adapter lengths were identified, resulting sequences were trimmed and re-aligned against the TKOv1 library with standard parameters on Bowtie2 5. Read count files for each condition were then re-generated with MAGeCK's (v0.5.9.3) count command using Bowtie aligned sequences. Variation in sgRNA read counts after treatment and individual gene level NormZ scores (Normalized Z-scores of up to 6 sgRNAs per gene) were obtained with the DrugZ algorithm (v1.1.0.2), a program developed for CRISPR screen analysis, running with default parameters (default parameters include: paired samples and a pseudocount of 5 added to each sgRNA count)
Data analysis	All data is representative of three or more independent experiments. For immunofluorescence studies, at least 100 nuclei were scored by each individual experiment. Prism ver 6.0 was used to do the statistical analyses (Ordinary one-Way ANOVA was used for statistical analysis of biochemical experiments and other experiments were analysed by two-tailed Student's t-test (Mann-Whitney test U-test)), unless mentioned otherwise in figure legends. The integrated intensity of individual BrdU foci and RPA2 foci were quantified by using GDSC ImageJ Find Foci plugins. Survival assays and nuclei counting were performed using the Gen5 Data Analysis Software v3.03.

For manuscripts utilizing custom algorithms or software that are central to the research but not yet described in published literature, software must be made available to editors and reviewers. We strongly encourage code deposition in a community repository (e.g. GitHub). See the Nature Portfolio guidelines for submitting code & software for further information.

Data

Policy information about availability of data

All manuscripts must include a data availability statement. This statement should provide the following information, where applicable:

- Accession codes, unique identifiers, or web links for publicly available datasets
- A description of any restrictions on data availability
- For clinical datasets or third party data, please ensure that the statement adheres to our policy

The source data and uncropped gel pictures will be provided as a source data file.

Field-specific reporting

Please select the one below that is the best fit for your research. If you are not sure, read the appropriate sections before making your selection.

☒ Life sciences ☐ Behavioural & social sciences ☐ Ecological, evolutionary & environmental sciences

For a reference copy of the document with all sections, see nature.com/documents/nr-reporting-summary-flat.pdf

Life sciences study design

All studies must disclose on these points even when the disclosure is negative.

Sample size	All data is representative of three or more independent experiments.
Data exclusions	Not applicable
Replication	Not applicable
Randomization	Not applicable
Blinding	Not applicable

Reporting for specific materials, systems and methods

We require information from authors about some types of materials, experimental systems and methods used in many studies. Here, indicate whether each material, system or method listed is relevant to your study. If you are not sure if a list item applies to your research, read the appropriate section before selecting a response.

Materials & experimental systems

n/a	Involved in the study	
☐	☒	Antibodies
☐	☒	Eukaryotic cell lines
☒	☐	Palaeontology and archaeology
☒	☐	Animals and other organisms
☒	☐	Human research participants
☒	☐	Clinical data
☒	☐	Dual use research of concern

Methods

n/a	Involved in the study	
☒	☐	ChIP-seq
☒	☐	Flow cytometry
☒	☐	MRI-based neuroimaging

Antibodies

Antibodies used	Antibodies used are listed in Suppl. Table 3 with the source and identifier
Validation	Antibodies are validate with Western blot or IF with tagged ectopic expression as positive control and siRNA knock down as negative control. For gH2AX, BrdU, cell with and without irradiation was used as positive and negative controls.

Eukaryotic cell lines

Policy information about cell lines

Cell line source(s)	All cell lines are listed in Material and Methods under the section cell lines

Authentication	All cell lines were authenticated by STR profiling at DFCI Molecular Diagnostics Laboratory
Mycoplasma contamination	cell line are all test for mycoplasma with PCR based mycoplasma detection method and only mycoplasma negative cell line were used in study. We test cell line in culture for mycoplasma monthly (e-myco mycoplasm PCR detection kit (25235 LiLif).
Commonly misidentified lines (See ICLAC register)	no commonly misidentified cell lines were used.
